# Refractive Error among Children Presenting to the Outpatient Department of Ophthalmology in a Tertiary Care Centre: A Descriptive Cross-sectional Study

**DOI:** 10.31729/jnma.8064

**Published:** 2023-03-31

**Authors:** Anjila Basnet, Rohit Pandit, Prabha Devi Chettri

**Affiliations:** 1 Department of Ophthalmology, KIST Medical College and Teaching Hospital, Mahalaxmi, Lalitpur, Nepal; 2 Department of Paediatrics, KlST Medical College anaTeaching Hospital, Mahalaxmi, Lalitpur, Nepal

**Keywords:** *children*, *ophthalmology*, *prevalence*, *refractive error*

## Abstract

**Introduction:**

Refractive error is an important component of the priority disease 'childhood blindness' within the vision 2020 initiative to eliminate avoidable blindness. Some 12.8 million in the age group 5-15 years are visually impaired from uncorrected or inadequately corrected refractive errors. Early detection and treatment of uncorrected refractive errors enable them to perform better in daily activities. This study aimed to find the prevalence of refractive error among children presenting to the outpatient Department of Ophthalmology in a tertiary care centre.

**Methods:**

A descriptive cross-sectional study was done among children at a tertiary care centre from 19 June 2021 to 25 December 2021 after receiving ethical approval from the Institutional Review Committee (Registration number: 2078/79/12). Children of the age group 6 to 15 years were included whereas those with other ocular problems such as corneal opacities, cataracts, ocular trauma, and conjunctivitis or submitted incomplete data forms were excluded from the study. Convenience sampling was used. Point estimate and 95% Confidence Interval were calculated.

**Results:**

Out of 239 children, 118 (49.37%) (43.03-55.71, 95% Confidence Interval) were found to have refractive error.

**Conclusions:**

The prevalence of refractive error among children was higher compared to other studies conducted in similar settings.

## INTRODUCTION

Refractive error is a state in which the optical system of a non-accommodating eye fails to bring parallel rays of light to focus on the retina. An estimated 153 million people over 5 years of age are visually impaired as a result of uncorrected refractive errors, of which 8 million are blind. Approximately 12.8 million children in the age group 5-15 years are visually impaired from uncorrected or inadequately corrected refractive errors, estimating a global prevalence of G.96%.^1^

Refractive error is an important component of the priority disease 'childhood blindness' within the vision 2020' initiative to eliminate avoidable blindness.^2^ Early detection and treatment of uncorrected refractive errors enable them to perform better in daily activities.

The study aimed to find out the prevalence of refractive error among children presenting to the outpatient Department of Ophthalmology in a tertiary care centre.

## METHODS

A descriptive cross-sectional study was conducted in the outpatient Department of Ophthalmology at KIST Medical College and Teaching Hospital from 19 June 2021 to 25 December 2021. Ethical approval was taken from the Institutional Review Committee (Registration number: 2078/79/12). The inclusion criteria for the study were children in the age group of 6-15 years. Children with other ocular problems such as corneal opacities, cataracts, ocular trauma, and conjunctivitis or submitted incomplete data forms were excluded from the study. Convenience sampling was used. The sample size was calculated using the following formula:


$$\begin{array}{ll} \text{n} & ={\text{Z}}^{2}\times \frac{\text{p}\times \text{q}}{{\text{e}}^{\text{2}}}\\ & =1.645^{2}\times \frac{0. 139\times 0.861}{0.05^{2}}\\ & =184 \end{array}$$

Where,

n = minimum required sample size
Z = 1.96 at 95 % Confidence Interval (CI)
p = prevalence of refractive error, 13.86^4^
q = 1-p
e = margin of error, 5%

The calculated sample size was 184. However, 239 samples were included in the study. Data regarding demographic details, previous spectacles use, and family history of refractive error were also collected. A detailed ocular examination was done including the recording of visual acuity by using Snellen's letter chart. The vision was measured with the child wearing their glasses if the child already had glasses on presentation. All children underwent the following examinations in the following sequence: visual acuity measurement of each eye separately (unaided and with a pin-hole), extraocular movement assessment, cover test, cover-uncover test, refraction using a Heine Beta 200 retinoscope, anterior segment examination with a slit lamp, and fundus examination using a direct ophthalmoscope or with +90D Volk lens. Cycloplegic refraction was done in all the cases of hypermetropia, scissor reflex, anisometropia, high refractive error, the cases where vision was not improved with normal refraction, and a suspected case of pseudomyopia. Cycloplegic refraction was done using 1% Cyclopentolate and 1% Tropicamide eye drops applied in a C-T-C pattern, with 5 minutes between applications (CTC protocol). Cycloplegia was considered complete if the pupils were dilated more than 6 mm. Refraction was performed using a streak retinoscope in a semidark room at a distance of 50 cm.

Refractive errors were classified according to the following definitions.^3^

1. Hyperopia: Refractive error at least +0.50 D. This was further classified as low (+0.50D to <+3.0 D), medium (+3.0 to <+6.0 D) and high hyperopia (+6.0 D or higher).
2. Myopia: Refractive error of at least -0.50D. This was further classified as low (-0.50 to <-3.0 D), medium (-3.0 to <-6.0D) and high myopia (-6.0 D or higher).
3. Astigmatism: Astigmatism was classified as simple hyperopic astigmatism, simple myopic astigmatism, compound hyperopic astigmatism, compound myopic astigmatism and mixed astigmatism.

Data was entered and analysed in Microsoft Excel 2016. Point estimate and 95% CI were calculated.

## RESULTS

Out of 239 respondents, 118 (49.37%) (43.03-55.71%, 95% CI) were found to have refractive error. Bilateral refractive error was 108 (91.53%) and the commonest type of refractive error was astigmatism 65 (55.08%) followed by myopia 37 (31.36%) and hypermetropia 16 (13.56%) (Table 1).

**Table 1:** Distribution and pattern of refractive error (n= 118).

Type of refractive error	n (%)
**Astigmatism**	
Compound myopic astigmatism	35 (29.66)
Simple myopic astigmatism	13 (11.02)
Mixed astigmatism	8 (6.78)
Simple hyperopic astigmatism	5 (4.24)
Compound hyperopic astigmatism	4 (3.39)
**Myopia**	
Low myopia	21 (17.79)
Medium myopia	13 (11.02)
High myopia	3 (2.54)
**Hypermetropia**	
Low hyperopia	11 (9.33)
Medium hyperopia	2 (1.69)
High hyperopia	3 (2.54)

The mean age of study participants was 9 years. Out of 118 respondents, the family history of refractive error was 46 (38.98%) and the most common age group was 6-9 years (Table 2).

**Table 2:** Socio-demographic variables of the participants (n= 118).

Variables	n (%)
**Age group (in years)**	
6-9	57 (48.31)
9-11	25 (21.19)
11-15	36 (30.51)
**Gender**	
Male	67 (56.78)
Female	51 (43.22)
Family history of refractive error	46 (38.98)
Father using glasses	21 (17.79)
Mother using glasses	25 (21.19)
Sibling using glasses	21 (17.79)
Previous use of glasses	68 (57.63)

## DISCUSSION

In this study the prevalence of refractive error was 49.37%, which was higher compared to similar studies conducted in India.^4^ Visual impairment from uncorrected refractive error can have immediate and long-term consequences in children and adults, such as lost educational and employment opportunities, lost economic gain for individuals, families, societies, and impaired quality of life. Various factors are responsible for refractive errors remaining uncorrected: lack of awareness and recognition of the problem at personal and family levels, as well as a community and public health level; non-availability of and/or ability to afford refractive services for testing; insufficient provision of affordable corrective lenses; and cultural disincentives to compliance. In the age group 5-15 years, noncorrection of refractive errors is due to several factors: the lack of screening and the availability and affordability of refractive corrections are the most important.^5^

Refractive error was most commonly seen in boys compared with girls which was similar to other studies.^4^ The prevalence of this study was similar to the study done in the Mechi zone of Nepal in which the prevalence of refractive errors was found to be 56%.^6^ A study conducted in Pakistan concluded with a prevalence of refractive error of 24.4%, myopia (52%) was the major type of refractive error followed by astigmatism (38.1%) and hypermetropia (9.8%).^7^

In the present study, astigmatism was 65 (55.08%) followed by myopia 37 (31.36%) and hypermetropia 16 (13.56%). Similarly, other studies also found that the maximum number of participants had a refractive error of astigmatism followed by myopia and hypermetropia.^3,8,9^

A study done in Africa concluded with the prevalence of refractive error was 11.6% which was lower than this study.^10^ Meanwhile, one study showed that positive family history, longer time spent on near-work activities and less outdoor activity were positively associated with myopia.^11^ Likewise in a study done in Nepal, the overall prevalence of refractive error was 9% which was lower than present study.^12^ In a study done in Iran, the prevalence of astigmatism was 16.7% which was almost similar to the present study.^13^

The limitation of this study was that the possible risk factors responsible for the development of the different types of refractive errors were not assessed. This was a hospital-based cross-sectional study. Therefore, the findings of the present study cannot be extrapolated to the entire population.

## CONCLUSIONS

The prevalence of refractive error among children was higher compared to other studies conducted in similar settings. Refractive errors in school children are major public health issues having many immediate and long-term consequences. Visual impairment due to uncorrected refractive errors will definitely jeopardise the achievement of two vital global indicators of development in the form of education and health. Public awareness, health education and school screening programs with periodic evaluation in the primary grades might be useful to identify and manage refractive error. School teachers; children and their parents should be educated about the signs and symptoms of refractive errors and the risk factors involved in their development.
